# Red Midge Larvae Are an Invertebrate Alternative Diet to Beef Liver for Planarian Husbandry

**DOI:** 10.3390/biom15121659

**Published:** 2025-11-27

**Authors:** Jonah Pacis, Danielle Ireland, Evangeline Coffinas, Jerome Sheehan, Kate Sun, Eva-Maria S. Collins

**Affiliations:** 1 Department of Biology, Swarthmore College, Swarthmore, PA 19081, USAdhagstr1@swarthmore.edu (D.I.);; 2 Department of Mechanical Engineering, Virginia Polytechnic Institute and State University, Blacksburg, VA 24061, USA; 3 Department of Physics and Astronomy, Swarthmore College, Swarthmore, PA 19081, USA; 4 Department of Neuroscience, Perelman School of Medicine, University of Pennsylvania, Philadelphia, PA 19104, USA; 5 Center of Excellence in Environmental Toxicology, University of Pennsylvania, Philadelphia, PA 19104, USA

**Keywords:** new approach method, *Dugesia japonica*, developmental neurotoxicology, behavior, diazinon, fluoxetine, bloodworms

## Abstract

Freshwater planarians are an emerging model for toxicology and neuroscience because of their amenability to rapid behavioral screening and remarkable ability to regenerate a cephalized nervous system. As invertebrates, planarians can help reduce the use of vertebrates in research; however, laboratories typically maintain planarians on diets of homogenized organic beef or chicken liver, raising ethical concerns with feeding a vertebrate diet. Organic liver is difficult to obtain, and preparation methods vary, introducing intra- and interlaboratory variability. Here, we show that *Dugesia japonica* planarians can be maintained for over a year on commercially available red midge larvae (RML), a natural prey of freshwater planarians. We found only minor effects on reproduction and gene expression. To explore dietary effects on behavior and chemical sensitivity, we compared the results of a chemical screen using dimethyl sulfoxide, diazinon, and fluoxetine on adult and regenerating *D. japonica*. We found that differences in potency and bioactivity for planarians on liver and RML diets were on par with inter-experiment variability of planarians fed the same diet. We also show that RNA interference is feasible with RML. Because RML requires no preparation and sustains planarian populations long-term, this invertebrate diet can substitute liver and help reduce the use of vertebrates in research.

## 1. Introduction

Freshwater planarians can regenerate their entire body from small tissue fragments, making them popular models for regeneration and stem cell biology [1]. Most remarkably, they can regenerate a cephalized nervous system that is compartmentalized with distinct neuronal cell populations and contains most of the same neurotransmitters as the mammalian brain [2,3,4]. In asexual planarians, neuroregeneration following asexual reproduction via binary fission is equivalent to neurodevelopment [4,5,6]. Because regeneration takes only 1–2 weeks [5,7], this invertebrate model allows for rapid studies of neurodevelopment [4]. Moreover, planarians are sensitive to their aquatic environment and react with stereotypical behaviors when exposed to certain chemicals [2,5,8,9,10], making them attractive models for pharmacology and toxicology. Recent reviews have discussed the history, challenges, and benefits of using planarians as a model for toxicology in general [11] and neurotoxicology, specifically [5].

Planarians ingest food via a specialized cylindrical eating tube, the pharynx, which they extend through a ventral opening to attach themselves to a food source [12]. The pharyngeal muscles then pull tissue and fluid from the food source via peristalsis [13,14]. Although planarians are carnivores known to feed on a variety of living or recently dead invertebrates, including annelids, mollusks, crustaceans, and insect larvae [15,16], laboratories typically rely on organic homogenized beef or chicken liver as a food source [17,18], which differ substantially from the natural diets of planarians. Liver introduces variability as labs source liver locally, leading to differences in nutritional composition, and may have different preparation methods, which can be labor-intensive [17]. Additionally, liver aliquots can only be stored up to 3 months at −20 °C (or 6 months at −80 °C), and liver stored longer than this can lead to bacterial infections and other planarian health problems [17]. Attempts to develop chemically defined diets for planarians have so far been unsuccessful in supporting long-term population growth [18], highlighting the need for an improved planarian diet. Ideally, this new diet would not be of vertebrate origin, in alignment with the aim to reduce, replace, and refine vertebrate animal use in research.

Here, we tested the hypothesis that *Dugesia japonica* (Ichikawa & Kawakatsu, 1964; Order: Tricladida, Family: Dugesiidae) planarians can be successfully maintained long-term on an invertebrate food source: the red aquatic larvae of non-biting midges (*Chironomus* sp.; Order: Diptera, Family: Chironomidae), also commonly referred to as bloodworms. Red midge larvae (RML) cohabitate with freshwater planarians in slow-moving bodies of water, and planarians are likely to encounter and feed on them in the wild [19]. In fact, one study proposed using planarians as a natural pesticide against RML because planarians are excellent predators [19]. Frozen, dead RML can be purchased in ready-to-use aliquots in bulk from commercial vendors. The ease of purchase and use of RML increases both accessibility to planarian research and decreases variability in diet between laboratories. The purpose of this study was to determine the suitability of an RML mono diet for planarian husbandry and to determine how diet affects planarian regeneration and reproduction, gene expression, and chemical susceptibility.

## 2. Materials and Methods

### 2.1. Planarian Species and Husbandry

The majority of experiments were performed with *D. japonica* planarians from an established laboratory culture that were cultivated in our lab for >1 year on their respective diets according to standard protocols [7,20]. Some experiments were also performed on *Dugesia dorotocephala* (Woodworth, 1987; Carolina Biological Supply Company, Burlington, NC, USA), asexual (strain CIWI4) or sexual *Schmidtea mediterranea* (Benazzi, Baguna, Ballester & del Papa, 1975; existing laboratory cultures), *Phagocata gracilis* (Haldeman, 1840; Ward’s Scientific, Rochester, NY, USA), and wild planarians caught from Crum Creek, Swarthmore, PA, that were cultivated in our lab for >1 year. Worms were initially stored in 0.5 g/L Instant Ocean (IO, Blacksburg, VA, USA) but were moved into 0.21 g/L Instant Ocean water with 0.83 mM MgSO_4_, 0.9 mM CaCl_2_, 0.04 mM KHCO_3_, and 0.9 mM NaHCO_3_ [21], which will be referred to as planarian water. Planarians were kept in BPA-free Tupperware containers (approximately 25 cm L × 14 cm L × 8 cm H) with the lid on loosely at 20 °C in a Panasonic refrigerated incubator in the dark. Planarians were fed twice a week with either organic beef liver purchased from a local butcher or commercially available red midge larvae (RML). The RML were bought directly from Brine Shrimp Direct (Ogden, UT, USA) and classified as *Chironomus* sp. (“frozen bloodworms”). RML were stored at −80 °C and sequentially moved to −20 °C as needed for use. Frozen RML were removed from their pre-aliquoted package, slightly crushed using a clean razor blade or plastic transfer pipette, and introduced into planarian containers with a plastic transfer pipette. Care should be taken when handling RML, as it has been reported that they can cause allergic reactions [22,23]. Planarian containers were cleaned approximately 2 h after each feeding and again 2 days later per standard protocols [24]. Planarians were fasted for at least 5 days before experimentation unless otherwise noted.

### 2.2. Regeneration Assays

Planarians raised on either liver or RML were fasted for 7 days and decapitated. Their head regeneration kinematics were quantified by measuring head width pre-amputation and blastema area on days 4–7 post-amputation as described in Campillo et al. [25]. Eight worms per condition were analyzed. A Student’s *t*-test was used to compare the average growth rate of planarian heads between the two diets.

### 2.3. Long-Term Population Growth Maintenance and Tracking

*Dugesia japonica* planarians of 7–9 mm in length were arbitrarily selected from a container of healthy specimens maintained on a liver diet and divided into two diet groups: liver and RML. Groups of 15 planarians were stored in individual containers in 200 mL of planarian water and stored in an 18 °C Panasonic incubator in the dark. Two independent populations were created for each diet group. Planarians were fed their respective diets of organic beef liver or frozen RML twice a week and cleaned five times a week: once before each feeding, once after each feeding, and then for another two days after the final weekly feeding. To ensure worms were consuming enough food, worms were fed for approximately 1–2 h until they were no longer interested in food. Planarian divisions were counted every 2 days. Tail pieces were removed from the population. One RML-fed worm became sick during the course of the experiment. This planarian was isolated in a separate petri dish and eventually died. This planarian was not replaced in the population.

#### 2.3.1. Imaging and Image Analysis of Planarians for Population Growth

Planarians were imaged weekly for ~1 year starting 86 days after the creation of the populations. Before each imaging session, the worm boxes were cleaned, and at least 5 representative, fully regenerated worms were transferred to petri dishes from either of the two replicate boxes. Petri dishes were placed onto an illuminated tracer panel with a visible ruler. The images were captured using an iPad once the worms began to glide and were fully elongated. The planarian area was measured using ImageJ software (Version v1.54p) [26] using a similar process as described in Campillo et al. [25].

#### 2.3.2. Statistical Analysis for Long-Term Population Growth

Statistical analysis was performed in R (Version 4.5.0) [27]. To examine the reproduction rate, the cumulative number of divisions over time was quantified. We modeled reproduction as a function of diet × time using a negative binomial generalized linear model with log link using the MASS package [28]. Statistical significance was assessed using Wald *z*-tests. *n* = 2 biological replicates (separate boxes) with 15 worms each. We modeled the area of individual worms as a function of diet × time with a Gaussian generalized linear model with identity link and assessed for statistical significance with a one-way ANOVA followed by Tukey’s Honestly Significant Difference Test. *n* = 5–18 biological replicates (individual worms) combined from the two boxes per diet for each timepoint.

### 2.4. Food Choice Assays

*Dugesia japonica* planarians, from populations that were fed either liver or RML for 9 months and fasted for 4 days, were manually selected to fall within a size range of 10 ± 2 mm. Four T-mazes were constructed on a 152 mm × 122 mm × 12.7 mm clear acrylic platform using a CNC milling machine based on a CAD model. Each T-maze was composed of a 60 mm stem and two 30 mm branches. T-mazes were filled with 3 mL of planarian water, and 6 worms were added to the base of each T-maze. First, two control runs were performed with each set of worms, where worms were allowed to glide within the maze for 5 min without any food present. Next, the planarians were removed from the T-maze, and RML and liver food pellets were submerged in separate arms of the T-maze. Creation of liver pellets was adapted from a standard protocol [29]. A protocol for RML pellet preparation is reported in the Supplementary Methods. Once food was added, the same six worms were added back to the base of the T-maze and allowed to move for 5 min, after which the number of worms in each section (left arm, middle, or right arm) was counted. Two independent experiments of six planarians each were performed for each diet. The location of each food source (i.e., left or right arm) was alternated between replicates. The number of worms that chose each food over the two independent experiments was summed, excluding planarians that did not make a choice (*n* = 1 per diet), leading to a total of *n* = 11 per diet group. For each diet group, to determine if one food was preferred over the other compared to random choice, a chi-squared test was performed in R (version 4.5.0, [27]).

### 2.5. RNA-Sequencing

#### 2.5.1. RNA Preparation and Sequencing

Planarians that had been maintained on their respective diet for over a year were fasted for 8 days prior to being frozen in −80 °C. One frozen *D. japonica* was homogenized per sample in TRIzol (Invitrogen, Waltham, MA, USA), and total RNA was extracted following the standard TRIzol Reagent Guide. Extracted RNA underwent DNase treatment using the TURBO DNase kit (Invitrogen) according to the manufacturer’s instructions. Three samples were prepared for each diet. The quality of the RNA extraction was first assessed on an agarose gel using an Agilent 2100 Bioanalyzer (Eukaryote Total RNA Nano kit; Agilent Technologies, Santa Clara, CA, USA) and confirmed via the TapeStation High Sensitivity D1000 Assay (Agilent Technologies), yielding RNA Integrity Numbers of 10 for all samples. Sequencing libraries preparation was performed by Admera Health (South Plainfield, NJ, USA) using the NEBNext Ultra II Directional with PolyA Selection kit (New England Biolabs, Ipswich, MA, USA). RNA-sequencing was performed by Admera Health using an Illumina NovaSeq X plus platform (San Diego, CA, USA). The prepared libraries were 150 base pair pair-end sequences with a read depth of 40 million pair-end reads per sample. The raw RNA-seq fastq files have been deposited on the NCBI Sequence Read Archive (BioProject ID PRJNA1367172).

#### 2.5.2. Bioinformatic Analysis

We utilized “https://scidap.com/ (accessed on 7 April 2025)”, a user-friendly bioinformatics platform for RNA sequencing data analysis. Since 80% of the *D. japonica* genome is made up of repeated sequences [30] and individuals accumulate an unusually large number of somatic mutations during asexual reproduction [31], establishing a reliable reference genome is extremely difficult. Although a whole-genome sequence of *D. japonica* was recently published [32], RNA-seq reads achieved greater mapping to a reference transcriptome [33], which was used for all downstream analyses.

The samples were run through the Kallisto transcript quant pipeline paired-end (version 5.0.0) workflow with default parameters through the SciDAP platform (Datirium LLC, Cincinnati, OH, USA). First, adapters were trimmed from the FASTQ files, and low-quality reads were filtered with TrimGalore. The filtered reads were pseudoaligned against the PlanMine 3.0 dd_Djap_v4 reference transcriptome [33], with gene annotations provided by SciDAP. Custom annotations were performed using BLASTx (National Center for Biotechnology Information, https://blast.ncbi.nlm.nih.gov/Blast.cgi?LINK_LOC=blasthome&PAGE_TYPE=BlastSearch&PROGRAM=blastx, accessed on 4 October 2023) against the UniProt database across various organisms. Filtering was performed to take the top hit based on bit score and only retain those with a bit score >50. Out of 74,909 transcripts, 66,499 had at least 1BLASTx result, and 28,896 remained after filtering. The output of BLASTx contained protein IDs, which were transformed to Gene and REFseq IDs using the ID mapping tool from UniProt (https://www.uniprot.org/id-mapping (accessed on 4 October 2023). The aligned reads were subsequently quality-assessed, sorted, and indexed.

Using the read counts output from the Kallisto transcript quant pipeline paired-end (version 5.0.0) workflow, quantitative differential expression analysis between liver-fed and RML-fed *D. japonica* was performed by the DESeq 11.0.0 pipeline workflow using default parameters through the SciDAP platform. Differentially expressed genes were defined as having log_2_ Fold Change (LFC) > ±0.59 and *p*_adjusted_ < 0.05. Using the GSEApy—Gene Set Enrichment Analysis in Python workflow (version 100) in SciDAP, we performed Gene Set Enrichment Analysis (GSEA) using the H_hallmark_gene_sets and default parameters.

### 2.6. Chemical Screening

#### 2.6.1. Chemical Preparations and Exposure

Chemical information is provided in Table 1. All chemicals were provided by Sigma–Aldrich (St. Louis, MO, USA). Stock solutions of diazinon and fluoxetine were prepared at 200× of the highest test concentration in dimethyl sulfoxide (DMSO), then diluted to the final 1× with planarian water right before exposure. As a solvent, DMSO was used at a final concentration of 0.5% in all test concentrations, a level which does not cause behavioral or morphological effects in *D. japonica* [34]. As a test chemical, DMSO was diluted directly in planarian water.

Planarians that had been fed on their respective diet for at least 6 months were used. Planarians were exposed to chemicals in a 48-well tissue culture-treated polystyrene plate (Genesee Scientific, San Diego, CA, USA), with each well containing 1 planarian in 200 μL of chemical solution. Adult and regenerating planarians were tested in separate plates. Regenerating planarians were amputated pre-pharyngeally with an ethanol-sterilized razor blade within 2 h of chemical addition.

All chemicals were screened over 5 concentrations (Table 1) plus a solvent control (0.5% DMSO or planarian water), with each row of the 48-well plate consisting of one concentration (*n* = 8). Plates were sealed with thermal film (Excel Scientific, Victorville, CA, USA) immediately after the addition of chemicals. Technical triplicates were run for all chemicals (total *n* = 24 per concentration), and following established protocols, a rotating orientation of chemical concentration in the plate rows was used to account for edge effects when screening [7]. Two independent experiments (*n* = 24 each) were conducted with planarians from each diet group to determine whether effects from diet were within the noise levels of repeated testing.

#### 2.6.2. Behavioral Screening

Effects on lethality and various planarian behaviors (locomotion, phototaxis, and noxious heat sensing) were assessed on days 7 and 12 of exposure using established procedures with a previously described automated screening platform [7,20,35,36]. These time points allow for evaluation of sub-chronic effects both during active regeneration (day 7) and following return to an adult-like state (day 12) [7] to determine the temporal effects of chemical exposure.

#### 2.6.3. Statistical Analysis for Chemical Screening

##### Lowest-Observed-Effect-Levels (LOELs)

Statistical analysis was performed on the compiled data of all individuals from the triplicate runs (*n* = 24) in R (Version 4.5.0) [27]. Each concentration was compared with its vehicle control population as previously described [7,37]. Except for lethality, only sublethal concentrations were analyzed. For lethality, stickiness, phototaxis, and scrunching endpoints, statistical significance was determined using a one-tailed Fisher’s exact test, with *p*-value adjustment for multiple testing using the Benjamini–Hochberg method. For speed and noxious heat sensing endpoints where sample distributions are normal, a Welch’s ANOVA was performed, followed by Tamhane–Dunnett’s post hoc test. For resting and anxiety, where the sample distribution is non-normal, statistical significance was determined by performing a Kruskal–Wallis test followed by a many-to-one Dunn’s post hoc test. For locomotor bursts where the sample distribution is over-dispersed, a negative binomial generalized linear model followed by a Dunnett’s post hoc test was used to determine statistical significance. LOELs indicate the lowest concentration with a statistically significant hit. We evaluated both all statistically significant hits and only those considered concentration-dependent by filtering out concentration-independent hits, which may not be biologically meaningful, to remove potential false positives. The compiled scores and their associated *p*-values are provided in File S1.

##### Benchmark Concentrations (BMCs)

As an alternative approach to determine hit calls and potency, we calculated the BMCs, a modeled concentration exceeding a defined benchmark response (BMR), for every chemical and readout. Readout scores were pre-processed and normalized as previously described [35,36] using MATLAB (R2024b; Mathworks, Natick, MA, USA). The preprocessed data were input into R (Version 4.5.0) [27], and BMCs were calculated using the Rcurvep package [38] as described in [36]. BMRs calculated from existing data from a large chemical library screen were used (Tables S1 and S2). BMCs are reported as the median BMCs calculated from *n* = 1000 bootstrapped concentration–response curves. The lower and upper limits (5th and 95th percentiles, respectively) of the BMC and associated hit confidence scores are listed in File S2. A readout was considered a hit if the hit confidence score exceeded 0.5. Some endpoints can have bidirectional changes in response, and thus, for these, BMCs were calculated for each direction separately.

### 2.7. RNA Interference (RNAi)

Double-stranded RNA was synthesized using the Invitrogen MEGAScript kit (Thermo Scientific, Waltham, MA, USA) as instructed in the manual. After the transcription step, the manual’s isopropanol precipitation protocol was utilized, though omitting the use of phenol/chloroform.

*Dugesia japonica* were fed liver or RML pellets containing 2 µg/µL *D. japonica* Transient Receptor Potential Ankyrin 1 (*Djtrpaa*) dsRNA or *Caenorhabditis elegans* (Maupas, 1900) *unc22* dsRNA, food coloring, and 2% agarose. Planarians were fed three times over the course of 7 days. To test for a knockdown, planarians were exposed to 100 μM Allyl isothiocyanate (AITC) (Sigma Aldrich, St. Louis, MO, USA), which induces scrunching through the DjTRPAa channel [39]. AITC was prepared fresh in planarian water. Worms were pipetted into a 100 mm petri dish containing 25 mL of 100 μM AITC solution. Five worms per condition were recorded at a time for bulk video recordings, and experiments were repeated 2 times. Image sequences were recorded with a FLIR Flea3 model FL3-U3-13E4M USB3 camera equipped with a 25 mm lens (Tamron, Edmund Optics, Barringtion, NJ, USA) using Point Grey FlyCapture 2 software. The camera was attached to a ring stand and recorded the planarians in the petri dish that was placed on an LED light panel (Tracer A4 LED Light Box, Amazon, Seattle, WA, USA).

## 3. Results

### 3.1. Planarians Can Be Maintained Long-Term on an RML Diet

**Figure 1:**
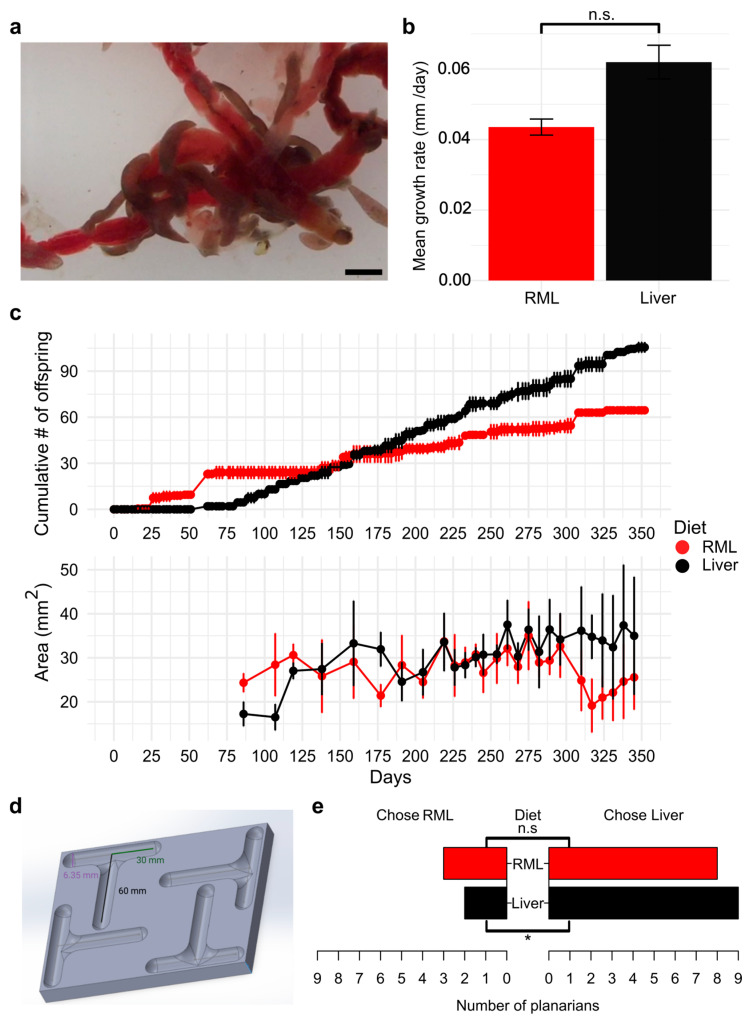
Long-term maintenance of *D. japonica* raised on an RML mono-diet is possible: (**a**) Image of *D. japonica* planarians ingesting RML. Scale bar: 2 mm. (**b**) Head regeneration kinematics of worms raised on their respective mono-diets. *n* = 8 for each diet. Error bars represent standard error. n.s.: not significant according to a Student’s *t*-test. (**c**) Planarian population growth by cumulative number of offspring (top) and individual worm growth by area (bottom). Data shown are the mean counts from two independent populations per diet (top) or the mean area of worms (*n* ≥ 5) imaged (bottom). Error bars represent standard deviation. (**d**) Schematic of T-maze design. Created in BioRender. Pacis, J. (2026) https://BioRender.com/kio7wqq. (**e**) Planarian food choice assay for worms raised on their respective mono-diets. *n* = 11 for each diet. One planarian from each diet group had “no choice” and was excluded from analysis for simplicity. * indicates statistically significant food preference (chi-squared test). n.s.: not significant.

We presented *D. japonica* planarians that were reared on liver for over a decade in our laboratory with commercially available frozen RML and found that they readily consumed RML (Figure 1a). Other planarian species *(Dugesia dorotocephala*, *Phagocata gracilis*, asexual and sexual *Schmidtea mediterranea*, and wild-caught planarians (Crum Creek, Swarthmore, PA, USA)) were also tested. We confirmed that the planarians consumed RML by checking worms for a dark red coloration that is visibly distinct from unfed planarians. All tested species readily consumed frozen RML (Figure S1a). Because of their dark black color, a color change could not be detected in *P. gracilis* planarians; thus, we confirmed they consumed RML through observation that this species’ many pharynges [40] were attached to the RML (Figure S1b). As we work primarily with *D. japonica* planarians, we focused on further studying the effects of diet in this species.

Next, we asked whether regeneration capacity or kinematics were affected by diet. We amputated the heads of planarians raised on the two diets and monitored their head regeneration over the course of a week. We found no significant difference in the rate of blastema regeneration between planarians raised on the two diets (*p* = 0.12) (Figure 1b).

Because other attempts at feeding alternative diets have not been successful for maintaining long-term growth in planarians [18], we first determined if *D. japonica* planarians could be successfully maintained for a year on an RML mono diet and compared the number of offspring produced on either an RML or liver diet (Figure 1c). A negative binomial generalized linear model revealed that the cumulative number of offspring increased over time in a diet-dependent manner (Diet × Days + 0.0062, *p* = 0.0079), with liver feeding having a significant effect on increased divisions as days passed. We observed no significant effect for diet type (*p* = 0.16) or days (*p* = 0.38) alone. In addition to counting the number of offspring produced, we imaged planarians weekly to determine whether there were any differences in size that could explain slower growth in the RML-fed worms. Previous studies showed that a threshold size is necessary for fission [41] and that worms that have a full gut do not divide as frequently [24]. Liver-fed worms showed a significant increase in size over time (Diet × Days interaction: +0.060 mm^2^, *p* < 0.001) while the same was not observed for RML-fed worms (*p* = 0.86). One-way ANOVA of deviance confirmed significant effects of diet (*F*_1,454_ = 64.14, *p* < 0.001), days (*F*_1,453_ = 37.83, *p* < 0.001), and their interaction (*F*_1,452_ = 41.08, *p* < 0.001), indicating that the growth rates were different between *D. japonica* fed on the two diets (Figure 1c). However, overall worm sizes were similar within biological and experimental noise, and the population growth of RML-fed worms remained sufficient for laboratory needs over the entire year.

### 3.2. Planarians Have a Mild Preference for Liver

Since planarians reproduce more quickly on a liver diet, we hypothesized that planarians prefer liver over RML when given a choice. To assess which food source planarians prefer, we presented planarians fed on their respective mono diets for nine months with a choice of either liver or RML using a custom T-maze (Figure 1d). Because liver disperses more rapidly in water and planarians display strong chemotaxis towards food, we prepared agarose pellets containing either homogenized liver or RML to standardize chemoattractant release (Supplementary Methods). In the absence of food, planarians randomly chose either arm of the T-maze (Figure S2). Chi-square analysis revealed that liver-fed planarians chose liver pellets significantly more than RML pellets (*p* = 0.03), while RML-fed planarians showed no significant differences in choice between the two foods (*p* = 0.13) (Figure 1e).

### 3.3. Gene Expression Differences Between Liver-Fed and RML-Fed D. japonica

**Figure 2:**
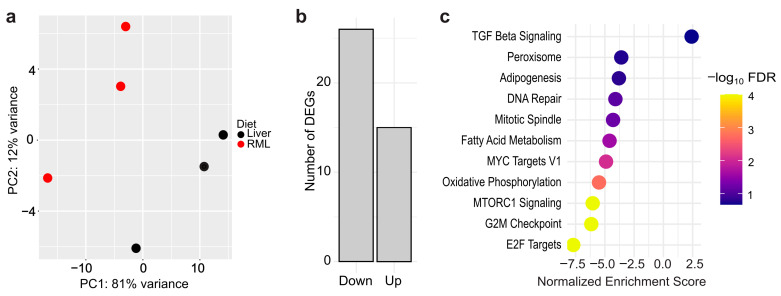
Dietary effects on gene expression are minimal between *D. japonica* planarians on a liver or RML mono-diet: (**a**) Principal component analysis plot based on regularized log_2_-transformed data. *n* = 3 biological replicates (1 worm per replicate). (**b**) Number of upregulated and downregulated differentially expressed genes (DEGs) in RML-fed worms compared to liver-fed worms. (**c**) Gene Set Enrichment Analysis for DEGs between worms raised on the two diets. Color bar corresponds to the negative log of the false discovery rate (FDR).

After sustaining *D. japonica* on an RML mono diet for over a year, we assessed whether dietary differences induced distinct gene expression using global RNA sequencing (RNA-seq) on three worms from each diet. Principal component analysis (PCA) showed separation along PC1 (81% variance) while modest separation was observed with respect to PC2 (12% variance) (Figure 2a). Differential gene expression analysis revealed that only 41 of 8740 genes (0.5%) identified by our RNA-seq experiment were differentially expressed (LFC > ±0.59, *p*-adj < 0.05) between liver-fed and RML-fed worms (File S3). Of the 41 differentially expressed genes (DEGs), 26 genes were downregulated while 15 were upregulated in RML-fed planarians (Figure 2b). Despite the small number of DEGs, we hypothesized that broader functional differences might still be present. Subsequently, we performed Gene Set Enrichment Analysis (GSEA) using the MSigDB Hallmark collection to identify enriched shifts in gene expression. This analysis revealed that 10 gene sets were enriched in RML-fed worms, with the greatest changes in E2F Targets, G2M checkpoint, and MTORC1 Signaling, and that one gene set, Transforming Growth Factor (TGF)-βeta Signaling, was enriched in liver-fed worms at a false discovery rate (FDR) <25% (Figure 2c, File S4).

### 3.4. RML-Fed Planarians Are Suitable for High-Throughput Chemical Screening

Because we had found some transcriptomic changes with the different diets, we sought to evaluate whether those diet-induced changes would impact sensitivity in response to chemical exposure. To this end, we screened three compounds: the solvent DMSO, which has been extensively tested in planarians [20,34,42,43]; diazinon, an organophosphorus pesticide that inhibits planarian cholinesterases and causes behavioral effects in adult and regenerating *D. japonica* [44], and fluoxetine, an antidepressant which we have found to cause behavioral effects in both acute [35] and chronic exposure in *D. japonica* [37]. Each chemical was tested at five concentrations in both intact/adult planarians and regenerating tail pieces. To determine whether differences between chemical screens of planarians fed liver in comparison to planarians fed RML were due to diet or due to inherent noise between screenings, we conducted two rounds of screening for both diets, corresponding to their ScreenID: Liver1, Liver2, RML1, or RML2. Liver1 and RML1 were performed simultaneously, while the other two screens were performed within several weeks of the first screen.

**Figure 3:**
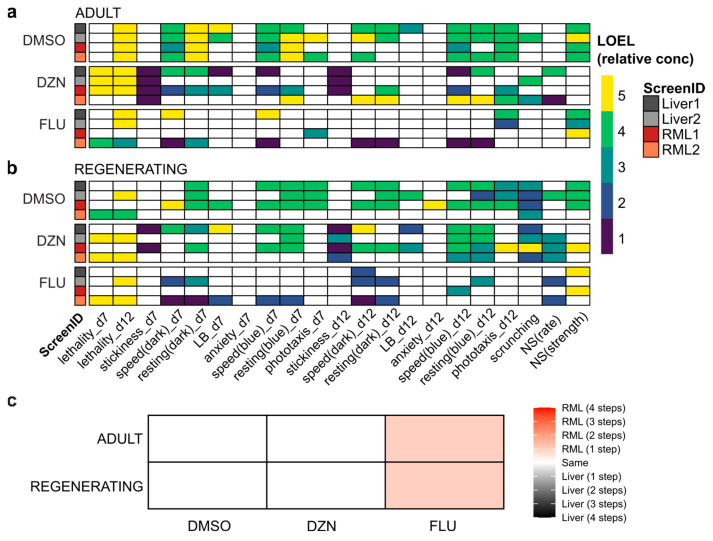
Behavioral screening results are robust to the new diet: (**a**,**b**) Heatmaps of effects of DMSO, diazinon (DZN), and fluoxetine (FLU) for all readouts with lowest-observed-effect-level (LOEL) denoted by color bar in relative concentration units (i.e., 1 is the lowest tested concentration and 5 is the highest) for (**a**) adult and (**b**) regenerating planarians on day 7 (d7) and day 12 (d12) of exposure. For all readouts besides lethality, only sublethal effects are shown. Only concentration-dependent hits are shown. LB: locomotor bursts; NS: noxious stimuli. (**c**) Matrix denoting planarian sensitivity to chemicals between diets. Sensitivity was calculated by LOEL across all behavioral readouts for each diet, chemical, and worm type (adult or regenerating). Color bar denotes the change in potency in terms of the number of concentration steps.

We compared the LOELs for the three chemicals for both adult (Figure 3a) and regenerating planarians (Figure 3b) across the different screens. All chemicals caused behavioral effects in adult and regenerating planarians at sublethal concentrations, in agreement with published data [20,34,36,37]. We observed variability in readout-specific bioactivity both across different diets, but also within the two experiments using the same diet. DMSO elicited the most robust per-readout results between individual screens, and fluoxetine yielded the most variable results.

We quantified readout concordance across screens as the occurrence of a statistically significant hit for the same chemical at the same behavioral readout in both screens or lack of effects in both screens (Figure S3a). Inter-screen variability was independent of diet. For example, both of the RML screens shared greater concordance with Liver1 and Liver2 than with each other in both adults and regenerating tails. Additionally, for regenerating tails, the Liver2 screen shared the greatest concordance with the RML2 screen, while the results of the Liver1 screen were most similar to RML1. The lowest per-readout concordance (39.7%) was observed for regenerating tails between RML1 and RML2. Fluoxetine was the primary driver for the low concordance, as seen in Figure 3b, which shows many hits for RML2 but only two hits for RML1. Notably, the same trend was true for fluoxetine when comparing Liver1 and two for regenerating tails. Readout concordance was even lower if concentration-independent hits were also included (Figures S3b and S4). While biologically plausible, concentration-independent hits are more likely to be false positives, which may reflect the greater variability seen across screens.

When considering the minimum LOEL across all readouts for both screens with each diet, RML-fed planarians showed increased sensitivity to fluoxetine at one lower concentration step than seen with liver-fed planarians (Figure 3c). For DMSO and diazinon, liver-fed and RML-fed worms displayed the same level of chemical sensitivity.

**Figure 4:**
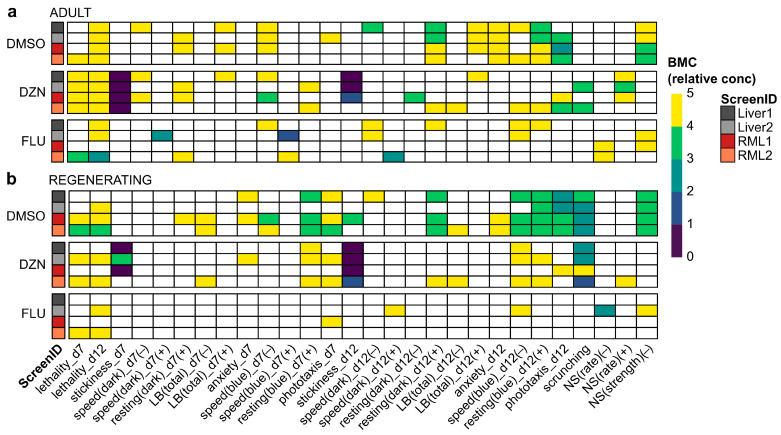
BMC analysis decreases noise in screening results: (**a**,**b**) Heatmaps of effects of DMSO, diazinon (DZN), and fluoxetine (FLU) for all readouts with BMC denoted by color bar in relative concentration units (i.e., 1 is the lowest tested concentration and 5 is the highest) for (**a**) adult and (**b**) regenerating planarians on day 7 (d7) and day 12 (d12) of exposure. Only readouts/directions with at least one hit across in any of the conditions are shown for simplicity. LB: locomotor bursts; NS: noxious stimuli.

Notably, because of the few chemicals tested in each condition for this screen, we could not apply biological relevance cut-offs as in our typical analysis pipeline [7]. Thus, the data shown in Figure 3 are based on statistical hits only, which we have shown are not robust to inter-worm variability [7]. Therefore, we also determined statistical significance and potency using BMC modeling. Unlike LOELs, which depend on the exact concentrations tested and do not consider concentration–response relationships, BMCs are calculated from regression analysis of the concentration–response curves and allow for the prediction of concentrations that will exceed a determined threshold (BMR). We used BMRs from a 112 chemical library screen that was conducted around the same time as this screen and verified that the distributions of control behaviors were not significantly different (Figure S5), justifying this approach. Because of the fixed threshold levels, BMCs allow for comparisons at the same effect level, whereas statistical significance can be achieved with different effect sizes depending on the variability of the control and test populations. BMC analysis generally identified fewer hits than LOEL analysis (Figure 4). This was especially true for regenerating planarians exposed to fluoxetine. This is because many of the low concentration hits seen with fluoxetine with LOEL analysis were hyperactive responses with flat or U-shaped response curves (Figure S6), which are difficult to pick up with the specific dose–response modeling used in this BMC analysis pipeline [38].

Readout concordance between screens was overall higher with BMC analysis than LOEL analysis, ranging from 69.7 to 85.9% in adult planarians and 74.7 to 87.9% in regenerating planarians (Figure S3c). As with LOEL analysis, the highest concordance was not always between screens of the same diet type. To compare potency differences across diets, we identified the minimum BMC (BMC_min_) across all readouts for each diet and worm type (Table 2). For DMSO and DZN, the BMC_min_ was the same or very similar in both diets. The BMC_min_ did differ considerably for fluoxetine across diets, with an order of magnitude difference in potency in regenerating tails. Notably, the increased fluoxetine sensitivity in the liver-fed regenerating tails was driven by a hit in a single readout (NS(rate) (+)) in the Liver2 screen.

### 3.5. The RML Diet Is Suitable for Mechanistic Studies in Planarians

Any new food source replacing liver needs to be amenable to RNA interference (RNAi) by feeding, as this is the primary mode of studying gene function in planarians [29]. Therefore, we tested the utility of the RML diet in RNAi applications by comparing the ability of RML pellets to deliver double-stranded RNA (dsRNA) and induce a well-defined knockdown phenotype comparable to standard liver pellets. Allyl isothiocyanate (AITC) is known to activate DjTRPAa, the planarian homolog of the Transient Receptor Potential Ankyrin 1 (TRPA1) in *D. japonica* [39]. When wildtype planarians are exposed, they elicit an escape response called scrunching, where the flatworm periodically changes its body via elongation and contraction cycles [45]. When TRPA1 is successfully knocked down, planarians will glide instead of scrunch when exposed to AITC [39,46]. We fed *D. japonica* planarians *Djtrpaa* dsRNA using either liver or RML pellets and compared the resulting behavioral responses to worms fed *unc-22* dsRNA, which targets a *C. elegans* gene with no planarian homolog. Behavioral recordings showed that both liver- and RML-pellets successfully induced *Djtrpaa* knockdown, as evidenced by RNAi planarians gliding in AITC solution (Video S1, Table 3).

## 4. Discussion

### 4.1. A RML Mono Diet Can Sustain D. japonica Planarians for at Least 1 Year with Minimal Physiological Changes Compared to a Liver Diet

Our findings demonstrate that RML are a viable alternative mono diet to liver for sustaining laboratory cultures of *D. japonica*. Other planarian species also readily consumed RML; however, their long-term response was not evaluated here. RML presents the benefits of a commercially available food source that is easy to acquire and requires no preparation. Another advantage of RML is that the husks that remain after feeding are easy to clean up and do not cloud the water as easily as liver remnants. Additionally, the replacement of a vertebrate diet with an invertebrate diet aligns with efforts in toxicology and pharmacology to replace mammalian-derived components, such as fetal bovine serum in in vitro studies [47], to increase sustainability and reproducibility. Bovine-derived products have been known to be subject to seasonal and geographic variation, introducing variability in experiments within and between laboratories [47].

*Dugesia japonica* were successfully maintained for 1 year on an RML mono diet with minimal physiological changes compared to liver-fed worms. RML-fed planarians retained the ability to fully regenerate their heads at similar rates to liver-fed planarians, supporting the use of RML-fed worms in regeneration studies. Future experiments to visualize planarian stem cell division post-feeding [18,48] using antibodies against phosphorylated histone 3 could be used to directly compare cell proliferation rates between worms raised on either diet. Using this approach, it was shown that significantly less stem cell proliferation was induced post-feeding in *S. mediterranea* planarians that consumed a defined diet made of synthetic components than when they consumed a liver diet, in alignment with the inability of the defined diet to maintain planarian growth long-term [18]. While liver-fed worms exhibited faster population growth (number of offspring over time), RML-fed individuals maintained stable sizes and reproduced successfully across a year-long period. Planarian size is highly variable, ranging from body lengths of approximately 0.5–20 mm, and is highly influenced by food intake, as starved planarians become smaller, through a process known as “degrowth” [49,50]. Thus, RML seem to contain sufficient nutritional value to maintain planarian growth. This is in contrast to findings with the synthetic diet in *S. mediterranea*, where planarians exhibited a gradual reduction in size and eventually developed lesions and died after 50 days of feeding [18]. The observation that RML-fed planarians divide less frequently may be explained if RML is less easily digested than liver, because having a full gut impedes fission [24]. In support of this idea, we observed two big increases in the number of offspring produced by RML-fed worms over the holidays, when planarians were fed and handled less frequently. Thus, the slower population growth seen in RML could possibly be mitigated by less frequent feedings. Another option is that RML are low on certain nutrients that facilitate growth and thus may need to be supplemented with additional invertebrate food sources to achieve the right nutritional balance.

Modest transcriptomic differences were found between planarians fed either diet. Only 0.5% of detected transcripts were differentially expressed between liver-fed and RML-fed worms, suggesting that global transcriptome profiles are largely conserved across these dietary conditions in *D. japonica*. In comparison, much larger transcriptomic differences were observed in developing zebrafish whose parents were fed either a commercial or a defined diet [51]. The small number of DEGs observed in this study should be interpreted in the context of several biological and technical limitations. First, planarians, including *D. japonica*, are not yet a fully established model system across molecular biology. Many species lack reliable reference genomes, and these genomes are not completely annotated. While a *D. japonica* reference genome has been published [32], our analyses achieved more robust mapping using a reference transcriptome [33], which may have limited our ability to detect all transcriptional changes across diets. Additionally, the GSEA hallmark gene sets are largely derived from human datasets and may not accurately reflect conserved biological function in planarians. Future studies that increase RNA-seq sample size from the *n* = 3 individuals per diet used here, expand functional annotations, and incorporate planarian-specific gene sets will provide a more complete picture of diet-dependent changes in planarians at the molecular level.

### 4.2. A RML Diet Is Suitable for Chemical Screening and Mechanistic Studies

It has been shown in various organismal models, including fruit flies [52], nematodes [53], and zebrafish [51,54,55,56], that diet can have a significant impact on important physiological phenotypes and toxicological outcomes, including health, morphology, behavior, stress response, and even mortality. Additionally, we have previously found that independent batches of liver caused substantial differences in chemical sensitivity in *D. japonica* planarians, despite no overt effects on the laboratory population [7]. Thus, we evaluated whether diet would impact chemical sensitivity or behavior using a proof-of-principle screen with three compounds that have previously been studied in *D. japonica*: DMSO, diazinon, and fluoxetine. Both adult and regenerating planarians raised on an RML diet responded to DMSO, diazinon, and fluoxetine with changes in behavior, with some differences in potency seen across diets. Other variables, such as chemical stock variability, variability in pipetting small volumes, worm size, time of screening, and food batch variability, could also affect inter-screen reproducibility. For example, Liver1 and RML1 were performed simultaneously using the same chemical stock solutions and were more similar to each other than to their respective diet replicate, which used independent chemical stock solutions. To contextualize the results and better understand how the differences we observed between the duplicate screens were affected by diet versus these other variables, we compared the results obtained here to published data from other screens.

DMSO has previously been tested in regenerating planarians using similar assays and LOEL analysis [20]. The current screen showed that regenerating planarians in both diets had LOELs of 0.5%, slightly lower than the previously reported 1% [20]. Notably, the more sensitive hits were only seen in one screen from each diet and were not detected with BMC analysis, which had a BMC_min_ of 0.79% in both diets, suggesting they may not be robust hits. Fluoxetine had previously been tested with the same behavioral platform using LOEL analysis [37]. We found that planarians raised on both diets showed increased sensitivity for adults in the current screen (LOEL of 0.1 (RML)/0.316 (liver) µM vs. 3.16 µM [37]) and comparable sensitivity for regenerating worms (LOEL of 0.1 (RML)/0.316 (liver) µM vs. 0.316 µM [37]) when only considering concentration-dependent hits. We also compared the results for diazinon from this screen to existing data using BMC analysis [36]. Previously, we found a BMC_min_ of 0.22 µM for adult planarians and 9.15 µM for regenerating planarians [36]. The values we found in the current screen are slightly higher for adult planarians but lower for regenerating planarians at 1 µM in both worm types, independent of diet type, with the caveat that we only tested to a minimum concentration of 1 µM in the current screen. Stickiness was the most sensitive readout in both these and the published data [36]. Thus, for all chemicals tested, inter-screen variability in chemical sensitivity is on par or higher than the variability induced by changing diets.

When looking at a per-readout basis, variability differed across readouts, chemicals, and statistical methods. Fluoxetine showed the greatest per-readout variability across the three chemicals tested, driven by the presence of hyperactive hits at low concentrations, which were not always detected in every screen, especially due to their non-monotonic dose response (compare Figure 3 and Figure S4). Notably low concentration hyperactive effects were also previously observed in regenerating planarians [37], and would be expected due to fluoxetine’s use as an antidepressant [43]. Thus, these hyperactive effects are not necessarily false positives but can be particularly challenging to identify robustly. This was especially true for BMC analysis, which is often less sensitive but provides greater confidence in hit detection [57]. As a result, readout concordance was much greater with BMC vs. LOEL analysis. We do not yet fully understand the biological significance of hits for individual readouts. Thus, in-depth analysis of readout information content and robustness will be necessary once the diet has been better controlled.

### 4.3. Further Considerations to Optimize Planarian Diet

The data presented here demonstrate that RML is a viable food source for long-term *D. japonica* planarian maintenance and is suitable for various types of planarian studies. Additionally, we showed that RNAi is possible using RML pellets and provided the protocol for this method in the Supplemental Methods so that other laboratories can also apply it. While this study shows that RML is a viable long-term diet for *D. japonica*, further investigation will be needed to determine if other planarian species can also be maintained long-term on a RML mono diet. Here, we only showed that they consume RML.

Our data from screening three chemicals (DMSO, diazinon, and fluoxetine) suggest that diet did not significantly impact chemical sensitivity. To build confidence that these findings are generalizable to other compounds, further comparative screens with more chemicals are warranted. Additionally, the feasibility of RNAi was shown using only one gene, *Djtrpaa*, which has a robust behavioral phenotype. We only confirmed knockdown through the behavioral phenotype and did not quantify knockdown efficiency by quantitative PCR. Thus, it is possible that the extent of gene knockdown differed between liver and RML pellets but was sufficient to induce the phenotype for this gene. Other phenotypes may be more sensitive to knockdown efficiency. Future research could extend to other genes to ensure the robustness of our results.

Organic liver was originally chosen as a food source for planarians because it is nutrient-rich and to reduce exposure to xenobiotics such as growth hormones, antibiotics, heavy metals, or pesticides. RML are typically farmed to be sold as fish food and thus are less regulated than food aimed at human consumption and may contain contaminants. For example, one study that investigated *Chironomidae* larvae from various sources (not used in this study) showed that some samples contained excess levels of lead [58], and various contaminants have been found in commercial fish food often used in zebrafish research [59]. Thus, a comparative analysis of the composition and identification of potential contaminants across various brands of RML will be necessary to identify the best source. Further, a mono diet consisting of RML may not be the most nutritionally balanced and may not be able to sustain planarian populations for decades, as is the case for liver. Thus, it is worth exploring additional invertebrate food sources, either to substitute RML or to be used in combination with RML. Ideally, one could fully control the planarians’ diet by feeding them a defined diet of synthesized food, based on an analysis of the nutrient composition of liver, as is currently being evaluated for many laboratory models [52,53,55,56,60]. While this has already been attempted in planarians, it has unfortunately not yet been successful for long-term planarian husbandry [18].

## 5. Conclusions

RML provide a practical and ethically preferable alternative to calf and chicken liver diets. To our knowledge, this is the first study to propose an alternative diet to liver that is able to sustain planarians long-term. The pilot screen from this study supports using an RML diet for toxicology studies and mechanistic research in *D. japonica*, reducing a barrier in culturing planarians and increasing accessibility of this emerging invertebrate model system. While liver remains the gold standard for maximum growth of laboratory cultures, we support and encourage the use of RML and the exploration of other invertebrate diets in laboratories seeking reproducible and ethically grounded research for planarians.

## Figures and Tables

**Table 1:** Chemicals screened in this study.

Chemical	CASRN	Purity	Tested Concentrations
Diazinon	333-41-5	98.0%	1, 3.16, 10, 31.6, 100 μM
Dimethyl sulfoxide (DMSO)	67-68-5	99.9%	0.1, 0.5, 1, 3, 4%
Fluoxetine hydrochloride	56296-78-7	98%	0.1, 0.316, 1, 3.16, 10 μM

**Table 2:** Most sensitive BMC per diet. The minimum BMC (BMC_min_) across all readouts and both screens for each diet is listed.

Planarian Type	Chemical	Diet	BMC_min_
Adult	DMSO	Liver	1.5%
	DMSO	RML	1.0%
	Diazinon	Liver	1.0 µM
	Diazinon	RML	1.0 µM
	Fluoxetine	Liver	0.15 µM
	Fluoxetine	RML	0.57 µM
Regenerating	DMSO	Liver	0.79%
	DMSO	RML	0.79%
	Diazinon	Liver	1.0 µM
	Diazinon	RML	1.0 µM
	Fluoxetine	Liver	0.57 µM
	Fluoxetine	RML	5.5 µM

**Table 3:** Penetrance of knockdown via RNAi using liver and RML pellets, respectively. A 100 µM AITC bath was used to induce scrunching. Scrunching was defined as 3+ consecutive scrunches. Two independent replicates with *n* = 5 worms for each condition were tested.

Diet	RNAi Population	# Planarians Scrunching/Total
liver	*unc22*	10/10
	*Djtrpaa*	1/10
RML	*unc22*	10/10
	*Djtrpaa*	0/10

## Data Availability

The original contributions presented in this study are included in the article/Supplementary Materials. Further inquiries can be directed to the corresponding author.

## Supplementary Materials

The following supporting information can be downloaded at: https://www.mdpi.com/article/10.3390/biom15121659/s1, Supplementary Methods: RML pellet preparation; Figure S1: Various species of planarians consume RML; Figure S2: Side preference of *D. japonica* planarians in a T-maze in the absence of food; Figure S3: Readout concordance across different statistical methods; Figure S4: Behavioral screening results are robust to the RML diet, when considering all hits; Figure S5: Benchmark responses (BMRs) are appropriate for this dataset; Figure S6: Example of fluoxetine non-monotonic dose response; Table S1: BMRs for binary endpoints; Table S2: BMRs for continuous endpoints; File S1: Compiled readout scores and *p*-values used for lowest-observed-effect-level analysis; File S2: Benchmark concentration (BMC), confidence intervals and hit scores for all readouts in adult and regenerating planarians from each screen; File S3: Output from DESeq2 analysis; File S4: Output from Gene Set Enrichment Analysis (GSEA); Video S1: Behavioral responses of *unc22* and *Djtrpaa* RNAi worms exposed to 100 μM AITC.

## Abbreviations

The following abbreviations are used in this manuscript:

NAM	New Approach Methodology
RML	Red Midge Fly Larvae
DMSO	Dimethyl sulfoxide
DZN	Diazinon
FLU	Fluoxetine
LOEL	Lowest Observed Effect Level
BMC	Benchmark Concentration
RNAi	RNA Interference
BMR	Benchmark Response
AITC	Allyl isothiocyanate
PCA	Principal Component Analysis
DEGs	Differentially expressed genes
FDR	False discovery rate
GSEA	Gene Set Enrichment Analysis
DjTRPAa	Dugeisa japonica Transient Receptor Potential Ankyrin 1
LB	Locomotor bursts
NS	Noxious stimuli

## References

[1] Ivankovic M., Haneckova R., Thommen A., Grohme M.A., Vila-Farré M., Werner S., Rink J.C. (2019). Model Systems for Regeneration: Planarians. Development.

[2] Buttarelli F.R., Pellicano C., Pontieri F.E. (2008). Neuropharmacology and Behavior in Planarians: Translations to Mammals. Comp. Biochem. Physiol. Part C Toxicol. Pharmacol..

[3] Cebrià F. (2007). Regenerating the Central Nervous System: How Easy for Planarians!. Dev. Genes Evol..

[4] Ross K.G., Currie K.W., Pearson B.J., Zayas R.M. (2017). Nervous System Development and Regeneration in Freshwater Planarians. Wiley Interdiscip. Rev. Dev. Biol..

[5] Ireland D., Collins E.S. (2023). Planarians as a Model to Study Neurotoxic Agents. Advances in Neurotoxicology.

[6] Ireland D., Collins E.-M.S. (2022). New Worm on the Block: Planarians in (Neuro)Toxicology. Curr. Protoc..

[7] Zhang S., Hagstrom D., Hayes P., Graham A., Collins E.-M.S. (2019). Multi-Behavioral Endpoint Testing of an 87-Chemical Compound Library in Freshwater Planarians. Toxicol. Sci..

[8] Venturini G., Stocchi F., Margotta V., Ruggieri S., Bravi D., Bellantuono P., Palladini G. (1989). A Pharmacological Study of Dopaminergic Receptors in Planaria. Neuropharmacology.

[9] Buttarelli F.R., Pontieri F.E., Margotta V., Palladini G. (2000). Acetylcholine/Dopamine Interaction in Planaria. Comp. Biochem. Physiol. Toxicol. Pharmacol. CBP.

[10] Rawls S.M., Patil T., Tallarida C.S., Baron S., Kim M., Song K., Ward S., Raffa R.B. (2011). Nicotine Behavioral Pharmacology: Clues from Planarians. Drug Alcohol Depend..

[11] Wu J.P., Li M.H. (2018). The Use of Freshwater Planarians in Environmental Toxicology Studies: Advantages and Potential. Ecotoxicol. Environ. Saf..

[12] Miyamoto M., Hattori M., Hosoda K., Sawamoto M., Motoishi M., Hayashi T., Inoue T., Umesono Y. (2020). The Pharyngeal Nervous System Orchestrates Feeding Behavior in Planarians. Sci. Adv..

[13] Ishii S., Sakurai T. (1991). Food Ingestion by Planarian Intestinal Phagocytic Cells—A Study by Scanning Electron Microscopy. Hydrobiologia.

[14] Forsthoefel D.J., Cejda N.I., Khan U.W., Newmark P.A. (2020). Cell-Type Diversity and Regionalized Gene Expression in the Planarian Intestine. eLife.

[15] Jennings J.B. (1962). Further Studies on Feeding and Digestion in Triclad Turbellaria. Biol. Bull..

[16] Vila-Farré M., Rink J.C., Rink J.C. (2018). The Ecology of Freshwater Planarians. Planarian Regeneration: Methods and Protocols.

[17] Dean M.R.P., Duncan E.M. (2020). Laboratory Maintenance and Propagation of Freshwater Planarians. Curr. Protoc. Microbiol..

[18] Abel C., Powers K., Gurung G., Pellettieri J. (2022). Defined Diets for Freshwater Planarians. Dev. Dyn..

[19] Legner E.F., Yu H.S., Medved R.A., Badgley M.E. (1975). Mosquito and Chironomid Midge Control by Planaria. Calif. Agric..

[20] Ireland D., Bochenek V., Chaiken D., Rabeler C., Onoe S., Soni A., Collins E.-M.S. (2020). *Dugesia japonica* Is the Best Suited of Three Planarian Species for High-Throughput Toxicology Screening. Chemosphere.

[21] Ireland D., Coffinas E., Rabeler C., Collins E.-M.S. (2025). Planarian Behavioral Screening Is a Useful Invertebrate Model for Evaluating Seizurogenic Chemicals. bioRxiv.

[22] Galindo P.A., Feo F., Gómez E., Borja J., Melero R., Lombardero M., Barber D., García Rodríguez R. (1998). Hypersensitivity to Chironomid Larvae. J. Investig. Allergol. Clin. Immunol..

[23] Meseguer Arce J., Villajos I.M.S.-G., Iraola V., Carnés J., Fernández Caldas E. (2013). Occupational Allergy to Aquarium Fish Food: Red Midge Larva, Freshwater Shrimp, and Earthworm. A Clinical and Immunological Study. J. Investig. Allergol. Clin. Immunol..

[24] Dunkel J., Talbot J., Schötz E.-M. (2011). Memory and Obesity Affect the Population Dynamics of Asexual Freshwater Planarians. Phys. Biol..

[25] Campillo N., Ireland D., Patel Y., Collins E.-M.S. (2023). A Simple Method for Quantifying Blastema Growth in Regenerating Planarians. Curr. Protoc..

[26] Schindelin J., Arganda-Carreras I., Frise E., Kaynig V., Longair M., Pietzsch T., Preibisch S., Rueden C., Saalfeld S., Schmid B. (2012). Fiji—An Open Source Platform for Biological Image Analysis. Nat. Methods.

[27] R Core Team (2021). R: A Language and Environment for Statistical Computing.

[28] Venables W.N., Ripley B.D. (2002). Modern Applied Statistics with S.

[29] Shibata N., Agata K., Rink J.C. (2018). RNA Interference in Planarians: Feeding and Injection of Synthetic dsRNA. Planarian Regeneration: Methods and Protocols.

[30] An Y., Kawaguchi A., Zhao C., Toyoda A., Sharifi-Zarchi A., Mousavi S.A., Bagherzadeh R., Inoue T., Ogino H., Fujiyama A. (2018). Draft Genome of *Dugesia japonica* Provides Insights into Conserved Regulatory Elements of the Brain Restriction Gene Nou-Darake in Planarians. Zool. Lett..

[31] Nishimura O., Hosoda K., Kawaguchi E., Yazawa S., Hayashi T., Inoue T., Umesono Y., Agata K. (2015). Unusually Large Number of Mutations in Asexually Reproducing Clonal Planarian *Dugesia japonica*. PLoS ONE.

[32] Tian Q., Guo Q., Guo Y., Luo L., Kristiansen K., Han Z., Fang H., Zhang S. (2022). Whole-Genome Sequence of the Planarian *Dugesia japonica* Combining Illumina and PacBio Data. Genomics.

[33] Rozanski A., Moon H., Brandl H., Martín-Durán J.M., Grohme M.A., Hüttner K., Bartscherer K., Henry I., Rink J.C. (2019). PlanMine 3.0—Improvements to a Mineable Resource of Flatworm Biology and Biodiversity. Nucleic Acids Res..

[34] Hagstrom D., Cochet-Escartin O., Zhang S., Khuu C., Collins E.-M.S. (2015). Freshwater Planarians as an Alternative Animal Model for Neurotoxicology. Toxicol. Sci..

[35] Ireland D., Rabeler C., Rao S., Richardson R.J., Collins E.-M.S. (2025). Distinguishing Classes of Neuroactive Drugs Based on Computational Physicochemical Properties and Experimental Phenotypic Profiling in Planarians. PLoS ONE.

[36] Ireland D., Zhang S., Bochenek V., Hsieh J.-H., Rabeler C., Meyer Z., Collins E.-M.S. (2022). Differences in Neurotoxic Outcomes of Organophosphorus Pesticides Revealed via Multi-Dimensional Screening in Adult and Regenerating Planarians. Front. Toxicol..

[37] Bayingana K., Ireland D., Rosenthal E., Rabeler C., Collins E.-M.S. (2023). Adult and Regenerating Planarians Respond Differentially to Chronic Drug Exposure. Neurotoxicol. Teratol..

[38] Hsieh J.H., Ryan K., Sedykh A., Lin J.A., Shapiro A.J., Parham F., Behl M. (2019). Application of Benchmark Concentration (BMC) Analysis on Zebrafish Data: A New Perspective for Quantifying Toxicity in Alternative Animal Models. Toxicol. Sci..

[39] Sabry Z., Ho A., Ireland D., Rabeler C., Cochet-Escartin O., Collins E.-M.S. (2019). Pharmacological or Genetic Targeting of Transient Receptor Potential (TRP) Channels Can Disrupt the Planarian Escape Response. PLoS ONE.

[40] Morgan T.H., Schiedt A.E. (1904). Regenerating in the Planarian *Phagocata gracilis*. Biol. Bull..

[41] Goel T., Ireland D., Shetty V., Rabeler C., Diamond P.H., Collins E.-M.S. (2021). Let It Rip: The Mechanics of Self-Bisection in Asexual Planarians Determines Their Population Reproductive Strategies. Phys. Biol..

[42] Stevens A.S., Pirotte N., Plusquin M., Willems M., Neyens T., Artois T., Smeets K. (2014). Toxicity Profiles and Solvent-Toxicant Interference in the Planarian *Schmidtea mediterranea* after Dimethylsulfoxide (DMSO) Exposure. J. Appl. Toxicol..

[43] Pagán O.R., Rowlands A.L., Urban K.R. (2006). Toxicity and Behavioral Effects of Dimethylsulfoxide in Planaria. Neurosci. Lett..

[44] Hagstrom D., Hirokawa H., Zhang L., Radic Z., Taylor P., Collins E.-M.S. (2017). Planarian Cholinesterase: In Vitro Characterization of an Evolutionarily Ancient Enzyme to Study Organophosphorus Pesticide Toxicity and Reactivation. Arch. Toxicol..

[45] Cochet-Escartin O., Mickolajczyk K.J., Collins E.-M.S. (2015). Scrunching: A Novel Escape Gait in Planarians. Phys. Biol..

[46] Arenas O.M., Zaharieva E.E., Para A., Vásquez-Doorman C., Petersen C.P., Gallio M. (2017). Activation of Planarian TRPA1 by Reactive Oxygen Species Reveals a Conserved Mechanism for Animal Nociception. Nat. Neurosci..

[47] Pfeifer L.M., Sensbach J., Pipp F., Werkmann D., Hewitt P. (2024). Increasing Sustainability and Reproducibility of in Vitro Toxicology Applications: Serum-Free Cultivation of HepG2 Cells. Front. Toxicol..

[48] Reddien P.W. (2018). The Cellular and Molecular Basis for Planarian Regeneration. Cell.

[49] Baguñà J., Romero R., Saló E., Collet J., Auladell C., Ribas M., Riutort M., García-Fernàndez J., Burgaya F., Bueno D., Marthy H.-J. (1990). Growth, Degrowth and Regeneration as Developmental Phenomena in Adult Freshwater Planarians. Experimental Embryology in Aquatic Plants and Animals.

[50] Thommen A., Werner S., Frank O., Philipp J., Knittelfelder O., Quek Y., Fahmy K., Shevchenko A., Friedrich B.M., Jülicher F. (2019). Body Size-Dependent Energy Storage Causes Kleiber’s Law Scaling of the Metabolic Rate in Planarians. eLife.

[51] Miller G.W., Truong L., Barton C.L., Labut E.M., Lebold K.M., Traber M.G., Tanguay R.L. (2014). The Influences of Parental Diet and Vitamin E Intake on the Embryonic Zebrafish Transcriptome. Comp. Biochem. Physiol. Part D Genom. Proteom..

[52] Lüersen K., Röder T., Rimbach G. (2019). Drosophila Melanogaster in Nutrition Research—The Importance of Standardizing Experimental Diets. Genes Nutr..

[53] Wang Y., Guo K., Wang Q., Zhong G., Zhang W., Jiang Y., Mao X., Li X., Huang Z. (2024). *Caenorhabditis elegans* as an Emerging Model in Food and Nutrition Research: Importance of Standardizing Base Diet. Crit. Rev. Food Sci. Nutr..

[54] Zhao W., Chen Y., Hu N., Long D., Cao Y. (2024). The Uses of Zebrafish (*Danio rerio*) as an in Vivo Model for Toxicological Studies: A Review Based on Bibliometrics. Ecotoxicol. Environ. Saf..

[55] Miller G.W., Labut E.M., Lebold K.M., Floeter A., Tanguay R.L., Traber M.G. (2012). Zebrafish (*Danio rerio*) Fed Vitamin E-Deficient Diets Produce Embryos with Increased Morphologic Abnormalities and Mortality. J. Nutr. Biochem..

[56] Fowler L.A., Williams M.B., Dennis-Cornelius L.N., Farmer S., Barry R.J., Powell M.L., Watts S.A. (2019). Influence of Commercial and Laboratory Diets on Growth, Body Composition, and Reproduction in the Zebrafish *Danio rerio*. Zebrafish.

[57] Ireland D., Word L.J., Collins E.-M.S. (2025). Statistical Analysis of Multi-Endpoint Phenotypic Screening Increases Sensitivity of Planarian Neurotoxicity Testing. Toxicol. Sci..

[58] Sharifian Fard M., Pasmans F., Adriaensen C., Laing G.D., Janssens G.P.J., Martel A. (2014). Chironomidae Bloodworms Larvae as Aquatic Amphibian Food: Bloodworms as Aquatic Amphibian Food. Zoo Biol..

[59] Tye M., Masino M.A. (2019). Dietary Contaminants and Their Effects on Zebrafish Embryos. Toxics.

[60] Zečić A., Dhondt I., Braeckman B.P. (2019). The Nutritional Requirements of *Caenorhabditis elegans*. Genes Nutr..

