# Supplementary material for: Red Midge Larvae Are an Invertebrate Alternative Diet to Beef Liver for Planarian Husbandry
Source: Biomolecules. 2025 Nov 27;15(12):1659. doi: 10.3390/biom15121659 (PMC12730930; doi:10.3390/biom15121659)
Supplement: Supplementary file 1 [file biomolecules-15-01659-s001.zip › SI files to submit/Supplemental Materials.pdf]

Red midge larvae are an invertebrate alternative diet to beef liver for planarian husbandry

Pacis, J., Ireland, D., Coffinas, E., Sheehan, J., Sun, K., Collins, E-M.S.

Supplementary Methods

RML pellet preparation

RML pellets were prepared as follows:

1. Prepare 2% low melt agarose: Dissolve 0.4 g low melting point agarose (Invitrogen, Carlsbad, CA) in Pearson water to a total volume of 20 mL. Microwave in 30s bursts until agarose is fully dissolved.
   a. Agarose can be aliquoted in 2 ml microcentrifuge tubes and aliquots can be stored for a few months at 4°C.
   b. If using previously made aliquots, heat the aliquots in a heat block at 60°C until the agarose is melted before using.
2. Thaw frozen RML cube for 5 minutes at room temperature.
3. Grind frozen RML into a homogeneous consistency with a porcelain mortar and pestle. There should not be large pieces of RML hull.
4. Transfer ground RML into a microcentrifuge tube and centrifuge at 21,130 x *g* for 30 seconds (Centrifuge 5424 with rotor FA-45-24-11, Eppendorf, Hamburg, Germany). Ground RMLs should separate into red supernatant and brown solids.
5. Pipet 86.25 μL of the RML supernatant (red liquid) using a P200 to a new microcentrifuge tube. Add dsRNA and food coloring here as desired following standard RNAi protocols for volumes [1].
6. Pipet 28.75 μL of 2% agarose to the centrifuge tube with supernatant. Vortex the contents. Briefly spin the mixture for 3-5 seconds in a tabletop microcentrifuge (BioExpress).
7. Pipet 10 μL pellets into a 100 mm petri dish. If being used for T-maze experiments, pellets can be placed on a strip of parafilm instead. Individual pellets (and the parafilm underneath) can be cut out of the parafilm strip in squares and placed directly in the T-maze. Mixture should make approximately 10 pellets. Cover the pellets with foil and store at -20 °C for maximum of 1 week.

Supplementary Figures

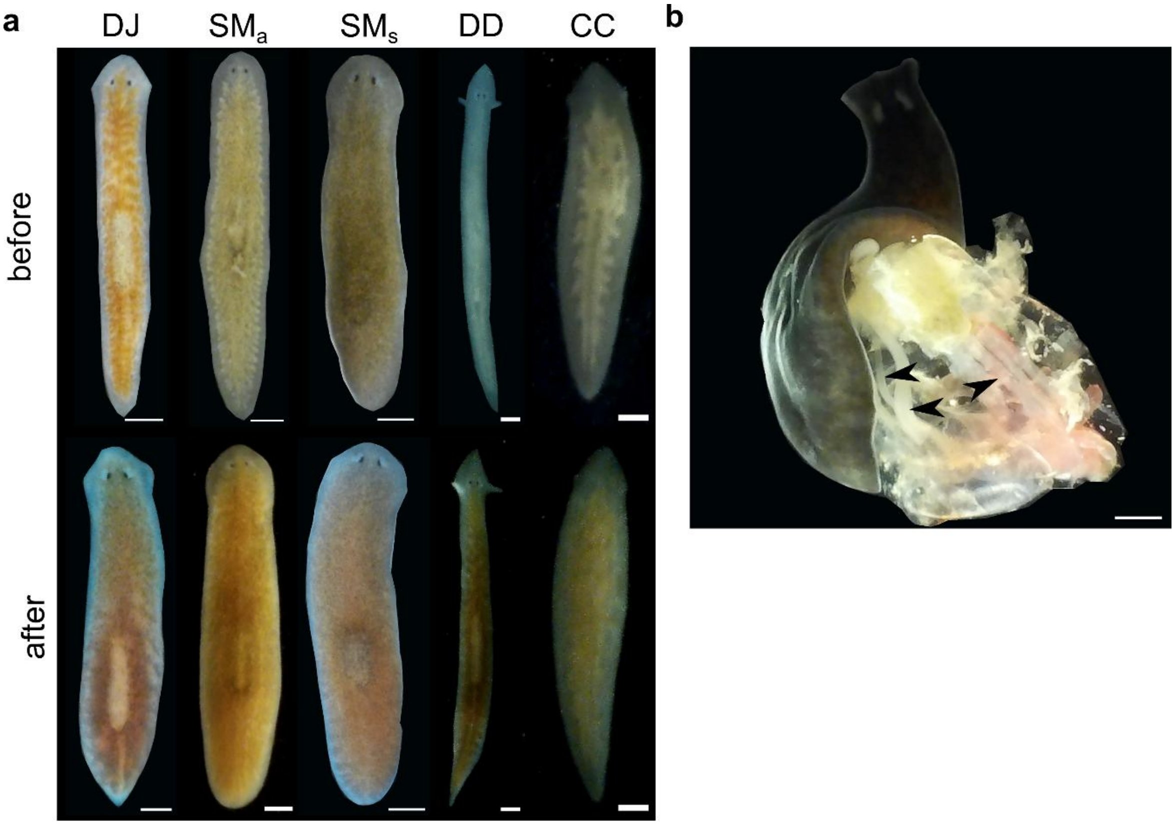

Figure S1. Various species of planarians consume RML. **a)** Representative images of *D. japonica* (DJ), asexual and sexual *S. mediterranea* (SM_a and SM_s, respectively), *D. dorotocephala* (DD), and wild planarians caught from Crum Creek, Pennsylvania (CC) photographed before and after a single feeding of RML. **b)** Image of a *Phagocata gracilis* planarian eating RML. This species of planarians can have multiple pharynxes [2] . A few pharynxes attached to the RML are indicated by arrow heads. Scale bars = 0.5 mm.

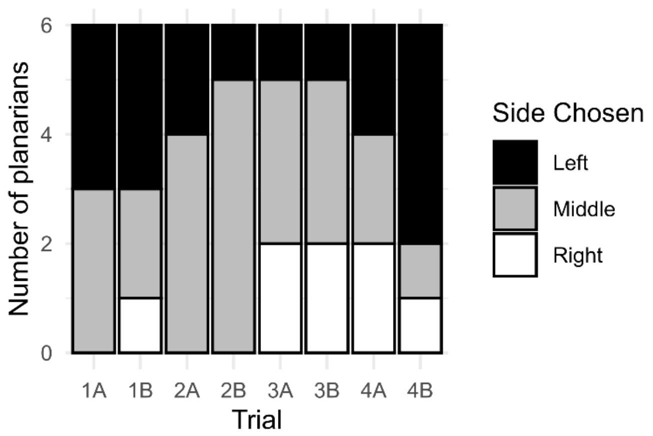

Figure S2. Side preference of *D. japonica* planarians in a T-maze in the absence of food. In the absence of food, planarians randomly enter the different areas of the T-maze. For each set of six worms tested in the food preference assay (Figure 1e), 2 control runs (A and B) in the absence of food were performed and the final location of each worm within the T-maze after 5 minutes is shown. Groups 1-2 are from liver-fed planarians, Groups 3-4 are RML-fed.

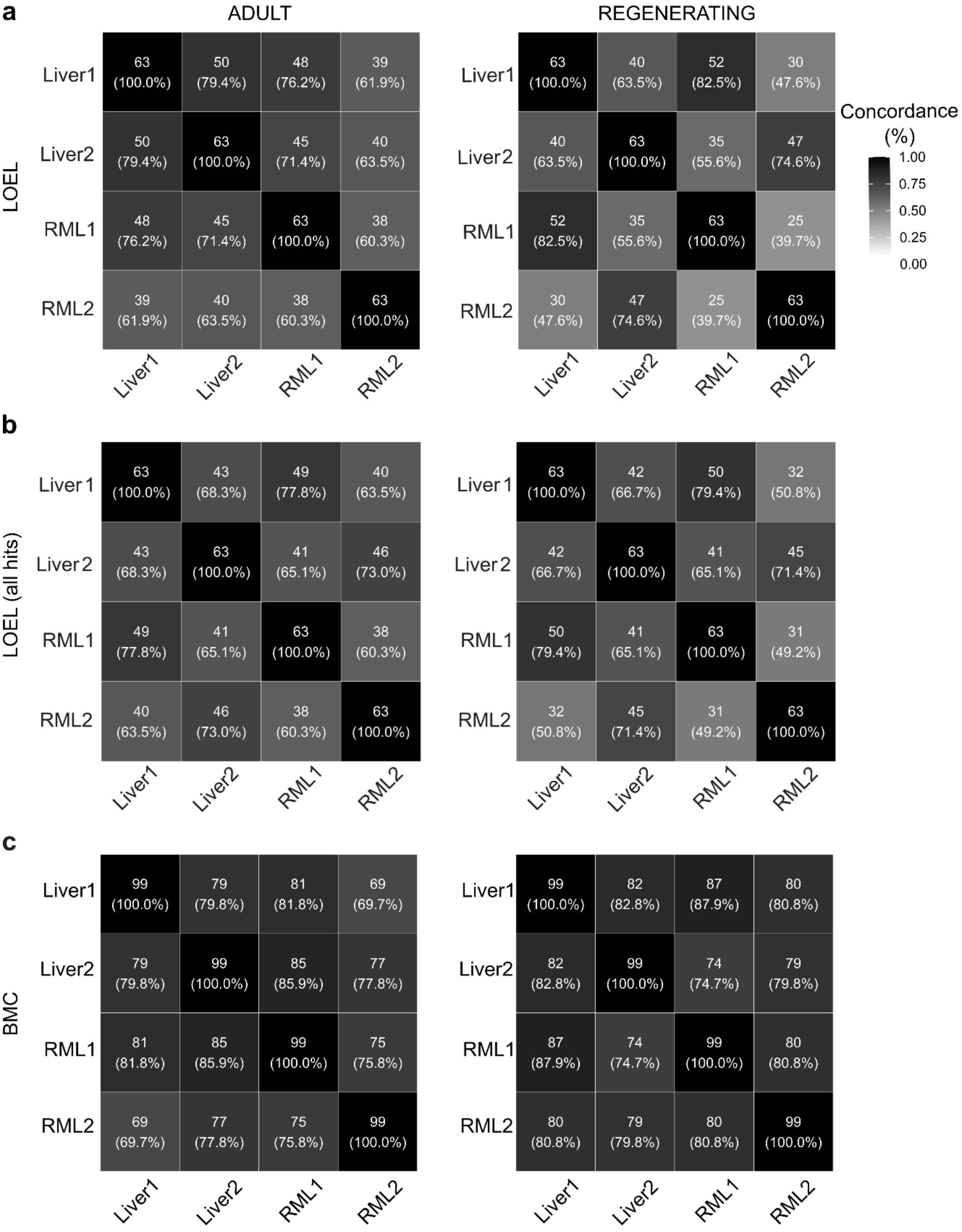

Figure S3. Readout concordance across different statistical methods. Concordance of hits between screens for adult (left) and regenerating planarians (right) when calculated via **(a)** Lowest-observed-effect-level (LOEL) considering only concentration-dependent hits, **(b)** LOELs considering all hits, and **(c)** BMC. Readouts are concordant across screens if there is a hit at any concentration level for the same

readout in both screens or no hit at that readout in both screens. Integers denote the number of concordant readouts between the two screens with the percent concordant noted in parenthesis.

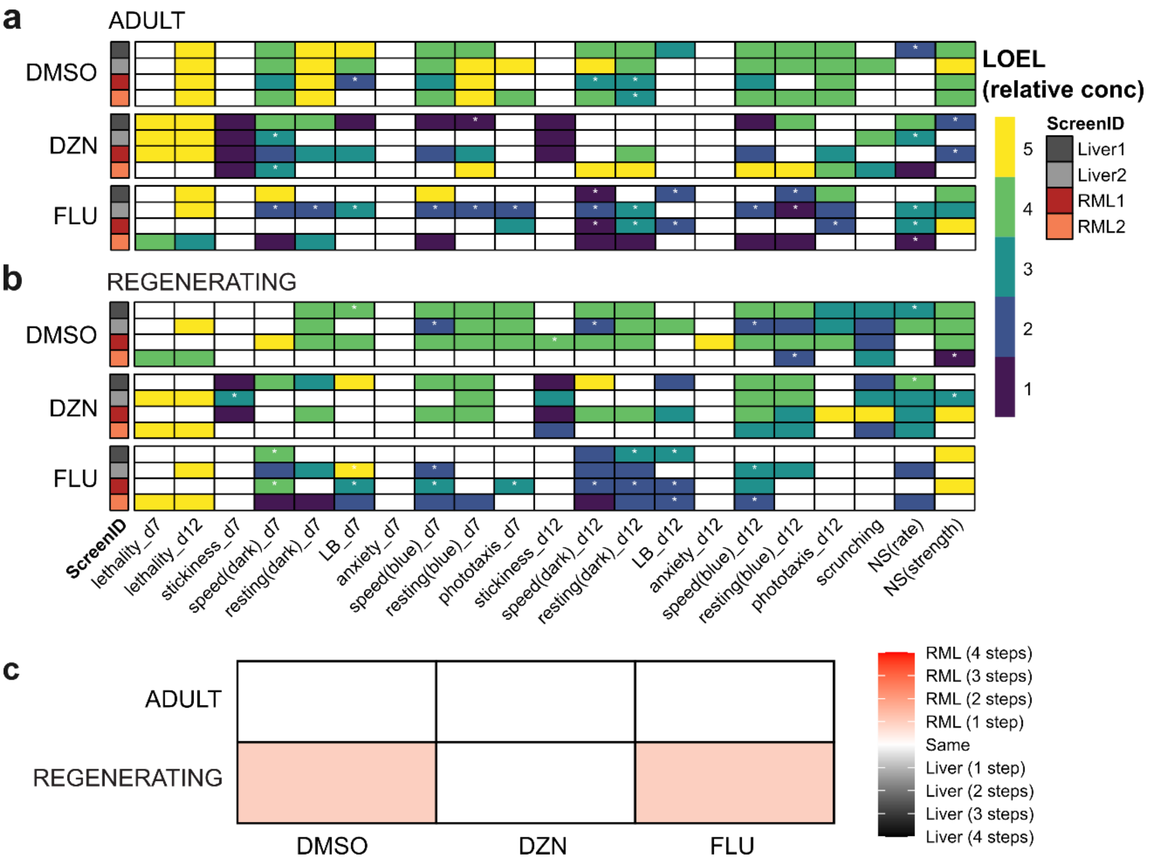

Figure S4. Behavioral screening results are robust to the RML diet, when considering all hits. **(a-b)** Heatmaps of effects of DMSO, diazinon (DZN), fluoxetine (FLU) for all readouts with lowest-observed-effect-level (LOEL) denoted by color bar for **(a)** adult and **(b)** regenerating planarians on day 7 (d7) and day 12 (d12) of exposure. For all readouts besides lethality, only sublethal effects are shown. Asterisks denote concentration-independent hits. LB: locomotor bursts; NS: Noxious stimuli. **(c)** Matrix denoting planarian sensitivity to chemicals between diets. Sensitivity was calculated as the LOEL across all behavioral readouts for each diet, chemical, and worm type (adults or regenerating). Color bar denotes the change in potency in terms of the number of concentration steps.

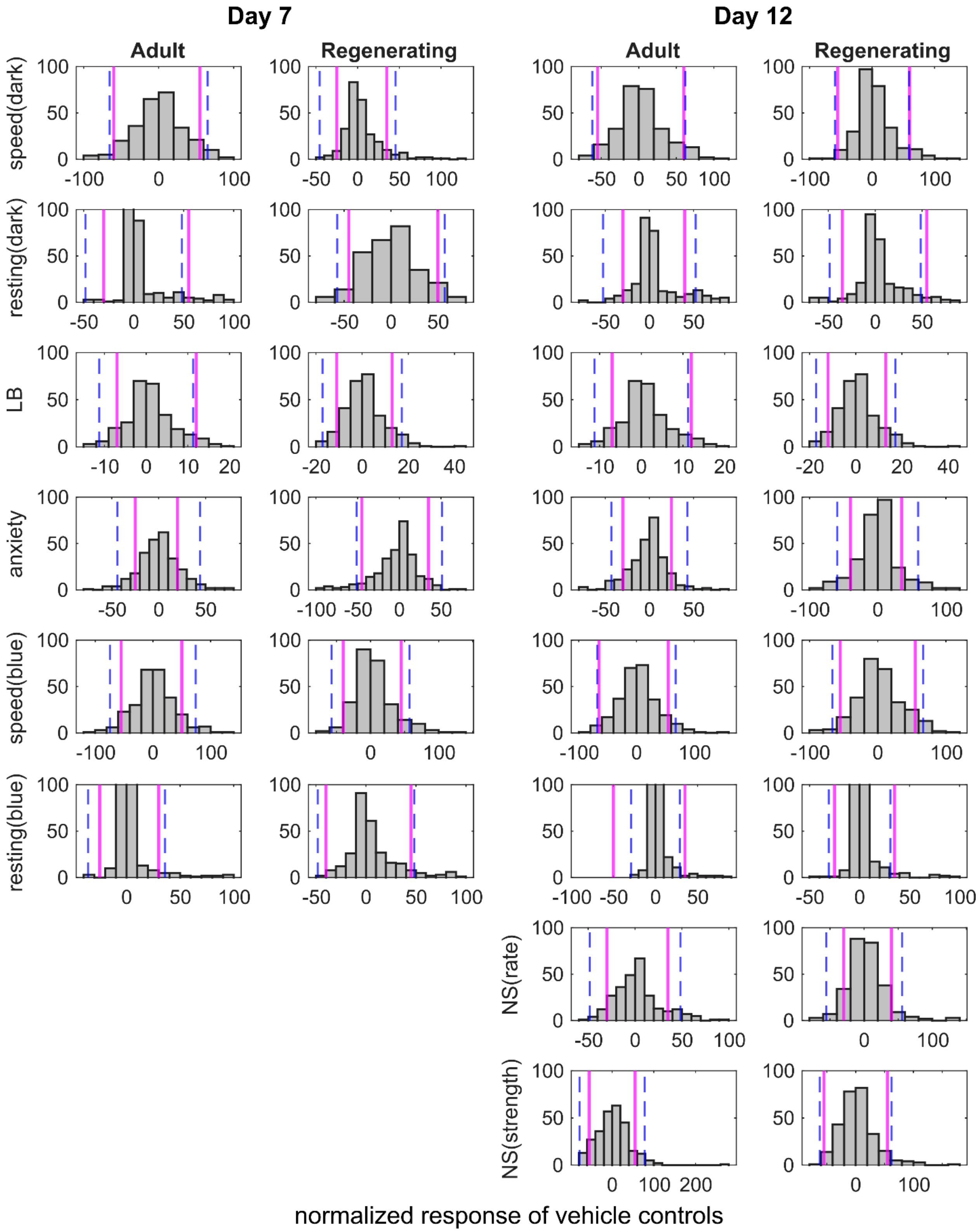

Figure S5. Benchmark responses (BMRs) are appropriate for this dataset. Histograms show the normalized responses of the vehicle controls from all chemicals and diets tested (n=288) for all continuous readouts. Blue dashed lines indicate -/+ 2 standard deviations. Pink lines indicate BMRs calculated from

an 112-chemical library screened at approximately the same time. The BMRs appropriately reflect responses outside of the majority of control behavior.

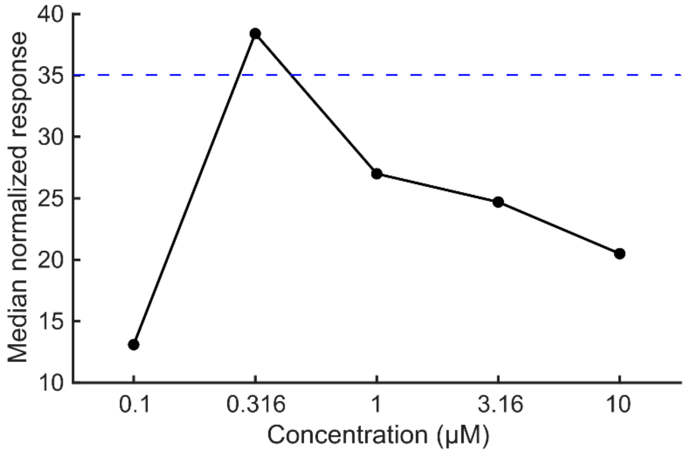

Figure S6. Example of fluoxetine non-monotonic dose response. The median normalized response for speed(dark)_d7 in fluoxetine treated regenerating planarians from the RML2 screen. LOEL analysis identified significant differences from 0.1 μM onwards while no hit was identified in BMC analysis. Although responses are seen above the BMR (blue dashed line), the inverse U-shape concentration-response curve likely precludes hit detection. Notably, the hit score is 0.467, just below the threshold of 0.5 to be counted as a hit.

Supplementary Tables

Table S1. BMRs for binary endpoints. Dashes indicate that the endpoint was not measured for that worm type and day.

Endpoint	Adult		Regenerating	
	D7	D12	D7	D12
Lethality	20	20	20	20
Stickiness	25	30	30	25
Phototaxis	35	30	30	30
Scrunching	--	30	--	30

Table S2. BMRs for continuous endpoints. For some endpoints, BMRs for both directions were determined and are listed in the order of increasing (+)/decreasing (-).

Endpoint	Normalization	Adult		Regenerating	
		D7	D12	D7	D12
Speed (dark)	$(Response_{chemical}-Response_{vehicle})$ *100	55/60	60/55	35/25	60/55
Resting (dark)	$(Response_{chemical}-Response_{vehicle})$ *100	55/30	40/30	50/45	55/35
Speed (blue)	$(Response_{chemical}-Response_{vehicle})$ *100	50/55	55/65	45/40	55/55
Resting (blue)	$(Response_{chemical}-Response_{vehicle})$ *100	30/25	35/50	45/40	35/25
Anxiety	$(Response_{chemical}/ Response_{vehicle})$*100-100	20/25	25/30	35/45	35/40
Locomotor bursts (total)	$Response_{chemical}-Response_{vehicle}$	12/7	12/7	13/11	13/12
Noxious stimuli (rate)	$(Response_{chemical}-Response_{vehicle})$ *100	--	35/30	--	40/30
Noxious stimuli (strength)	$(Response_{chemical}/ Response_{vehicle})$*100-100	--	55/55	--	55/55

Supplementary Files

File S1. Compiled readout scores and p-values used for lowest-observed-effect-level analysis.

File S2. Benchmark concentration (BMC), confidence intervals and hit scores for all readouts in adult and regenerating planarians from each screen. The BMC is listed as BMC_median. BMC_cil and BMC_ciu represent the lower and upper confidence intervals, respectively. Hit_confidence scores greater than 0.5 were considered hits. Some readouts were assessed in both increating (+) and decreasing (-) directions and are listed separately. Concentrations are listed in % for DMSO and µM for diazinon (DZN) and fluoxetine (FLU).

File S3. Output from DESeq2 analysis.

File S4. Output from Gene Set Enrichment analysis (GSEA).

Video S1. Behavioral responses of *unc22* and *Djtrpaa* RNAi worms exposed to 100 µM AITC.

Supplemental References

1. Shibata, N.; Agata, K. RNA Interference in Planarians: Feeding and Injection of Synthetic dsRNA. *Methods in Molecular Biology* **2018**, *1774*, 455–466, doi:10.1007/978-1-4939-7802-1_18/COVER.
2. Thomas Hunt Morgan; Schiedt, A.E. Regenerating in the Planarian Phagocata Gracilis. *Biological Bulletin* **1904**, *7*, 160–165.
